# Construction and validation of a prediction model of extrahepatic metastasis for hepatocellular carcinoma based on common clinically available data

**DOI:** 10.3389/fonc.2022.961194

**Published:** 2022-11-16

**Authors:** Liuxin Zhou, Li Ren, Wenhao Yu, Mengjian Qi, Jiaqi Yuan, Wen Wang, Xiaoxia Su, Fengjiao Yin, Manjun Deng, Haijiu Wang, Hongmu Long, Jiangchao Zeng, Jiajian Yu, Haining Fan, Zhixin Wang

**Affiliations:** ^1^ Department of Hepatopancreatobiliary Surgery, The Affiliated Hospital of Qinghai University, Xining, Qinghai, China; ^2^ Department of Hepatopancreatobiliary Surgery, The Chongqing University Fuling Hospital, Fuling, Chongqing, China

**Keywords:** primary hepatic carcinoma, extrahepatic metastases, risk factors, clinical features, the prediction model

## Abstract

**Objective:**

This study aimed to investigate the clinical characteristics and risk factors of patients with hepatocellular carcinoma (HCC) with extrahepatic metastases (EHM) and to establish an effective predictive nomogram.

**Methods:**

Clinical and pathological data from 607 patients with hepatocellular carcinoma admitted to the Affiliated Hospital of Qinghai University between 1 January 2015 and 31 May 2018 were documented, as well as demographics, clinical pathological characteristics, and tumor-related parameters to clarify clinical risk factors for HCC EHM. These risks were selected to build an R-based clinical prediction model. The predictive accuracy and discriminating ability of the model were determined by the concordance index (C-index) and the calibration curve. The results were validated with a bootstrap resample and 151 patients from 1 June 2018 to 31 December 2019 at the same facility.

**Results:**

In multivariate analysis, independent factors for EHM were neutrophils, prothrombin time, tumor number, and size, all of which were selected in the model. The C-index in the EHM prediction model was 0.672 and in the validation cohort was 0.694. In the training cohort and the validation cohort, the calibration curve for the probability of EHM showed good agreement between the nomogram prediction and the actual observation.

**Conclusion:**

The extrahepatic metastasis prediction model of hepatocellular carcinoma constructed in this study has some evaluation capability.

## Introduction

Hepatocellular carcinoma (HCC) is the leading malignant tumor from the liver, which is the seventh most common, and has the second highest death rate (in all 36 tumors) (1). It has already been reported in the article that the risk factor for poor prognosis is the lack of diagnosis of extrahepatic metastasis (EHM) (2). Therefore, an accurate evaluation of HCC metastases is critical for improved prognosis.

HCC is a kind of cancer that develops as a result of a secondary liver illness [such as viral hepatitis (HBV or HCV), alcoholic or fatty liver disease]. Liver function indices are intimately linked to the occurrence and development of HCC (3, 4). Besides, studies have reported that primary tumor progression characteristics (such as vascular invasion, tumor size and number, etc.) are independent risk factors for EHM (5, 6). The above parameters can be risk factors for metastatic HCC and will be included in this study as observational information. Since patients with HCC usually receive antitumor treatment during the clinical course, it may give a confounding effect on the analysis of metastatic factors. As a result, we evaluated the clinical features and risk factors of patients with HCC and EHM who did not receive anti-tumor therapy, and we created an effective EHM diagnostic nomogram.

## Patients and methods

### Patients and study design

A retrospective study was conducted on patients who were diagnosed with HCC from 1 January 2015 to 31 December 2019 at the Affiliated Hospital of Qinghai University (Xining, China). Inclusion criteria were as follows: 1) according to the Guidelines for diagnosis and treatment of primary liver cancer, the patient was diagnosed with HCC (2021 Edition) (7) and 2) with a complete medical record. Exclusion criteria were as follows: 1) no prior history of anticancer treatment; 2) no priors for other cancers; and 3) without other confirmed or suspected cancers. The training cohort consisted of patients between 1 January 2015, and 31 May 2018, and the validation cohort consisted of patients between 1 June 2018 and 31 December 2019. Depending on whether EHM was present at the time of the first diagnosis, the training cohort was further split into extrahepatic metastatic (observation group) and non-extrahepatic metastatic (control group) groups. Age and sex-related demographic data as well as clinicopathological characteristics such as body mass index, smoking and drinking history, blood tests, assessments of HBV and HCV infections, results of liver function tests, and tumor-related parameters were prospectively gathered.

### Diagnosis and definitions

The appearance of a newly detected tumor confirmed on two radiologic images, with or without an elevation of serum tumor markers, was defined as metastasis. A patient with a smoking history was defined as having smoked continuously or cumulatively for 6 months or more in the past. Drinking more than three standard glasses of alcohol per day or more than seven standard glasses of alcohol per week for 1 month or more, either continuously or cumulatively, is considered a drinking history.

### Follow-up

During the 2 years following diagnosis, all patients were seen once every 3 months. An abdominal ultrasound, blood test, and liver function test were performed at each of the follow-up visits. When a tumor recurrence or metastasis was suspected, a contrast-enhanced CT or MRI was performed, and the results were reviewed individually by two experienced doctors.

### Statistical analyses

Statistical analyses to identify risk factors were performed using IBM SPSS Statistics 23.0 for Windows (SPSS Inc., Chicago, IL, USA). Continuous variables were compared using the Mann–Whitney *U* test for variables with an abnormal distribution. Categorical variables were compared using the chi-squared or Fisher exact test. In the univariate analyses, p < 0.05 was considered statistically significant. Multivariate logistic regression analysis was used to evaluate the independent risk factors of extrahepatic metastases. In the multivariate analyses, p < 0.05 was considered statistically significant.

**Figure 1:**
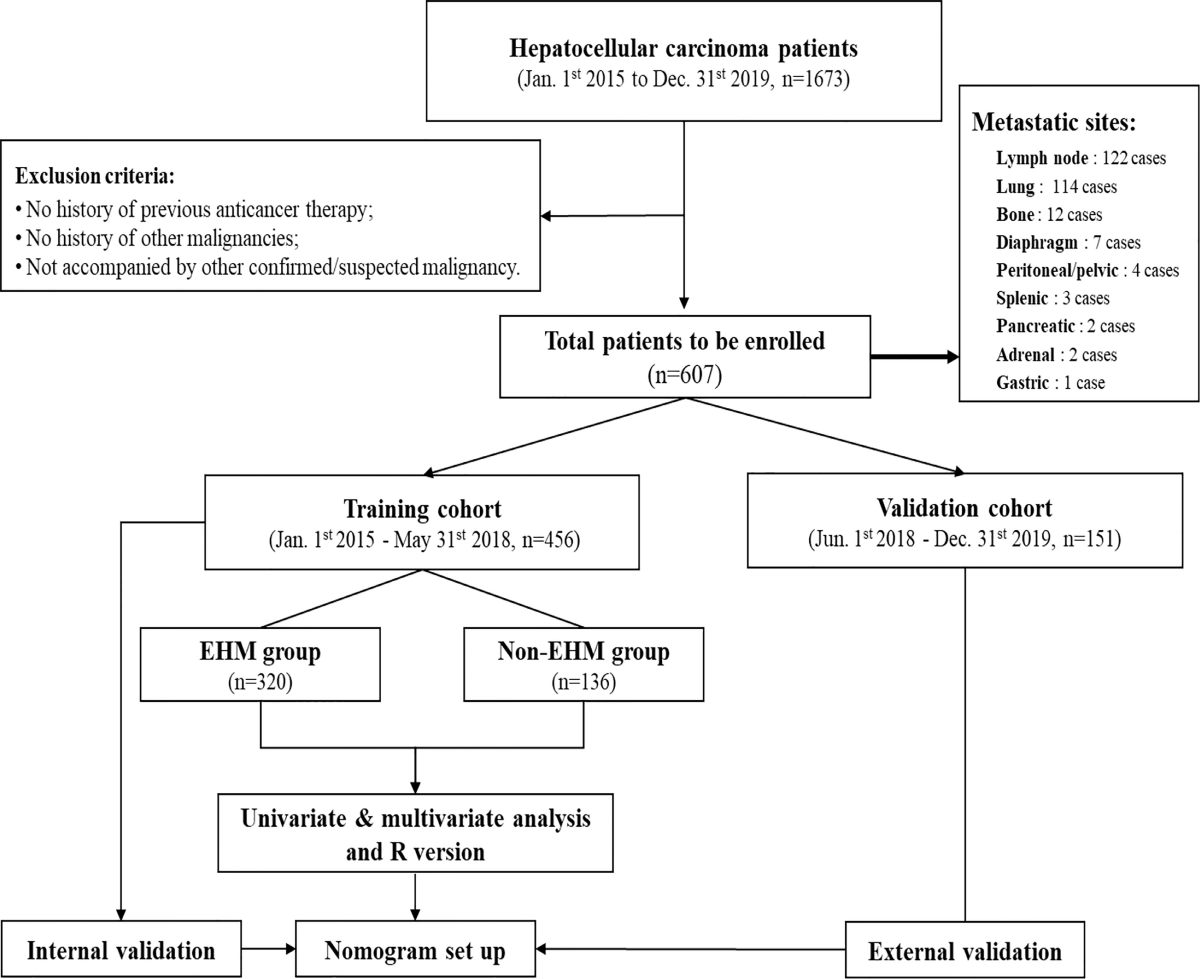
After the screening of medical records for the study, 607 cases were identified for final enrolment based on exclusion criteria. The training cohort was used to identify factors that were able to predict EHM, thereby establishing a nomogram for this study. This nomogram was then validated regarding its accuracy in the evaluation of EHM risk by using both the training cohort and validation cohort.

A nomogram was formulated based on the results of multivariate logistic regression analysis and by using the ‘rms’ package in R version 4.12 (http://www.r-project.org/). A final model selection was performed by a forward conditional selection process. The predictive performance of the nomogram was measured by concordance index (C-index) and the calibration curve. Bootstraps with 1000 resample were used for these activities (**Figure 1**).

## Results

### Presentation of patients

In this study, during the defined study period (January 1st,2015 to December 31st,2019), 1673 cases were identified as HCC in the Affiliated Hospital of Qinghai University. 1066 cases were excluded according to the exclusion criteria, and 607 cases were finally enrolled, including 456 patients in the training cohort from 1 January 2015 to 31 May 2018, and 151 patients in the validation cohort from 1 June 2018 to 31 December 2019 (**Figure 1**). Among all the 170 EMH patients enrolled at the time of diagnosis, 122 patients (71.8%) had lymph node metastasis, 114 patients (67.1%) had lung metastasis, 12 patients (7.1%) had bone metastasis, 7 patients (4.1%) had diaphragm metastasis, 4 patients (2.9%) had peritoneal or pelvic metastasis, 3 patients (2.2%) had splenic metastasis, and pancreatic metastases in 2 cases (1.5%), adrenal metastases in 2 cases (1.5%) and gastric metastases in 1 case (0.8%).

### Baseline of characteristics and multivariate analysis

**Figure 2:**
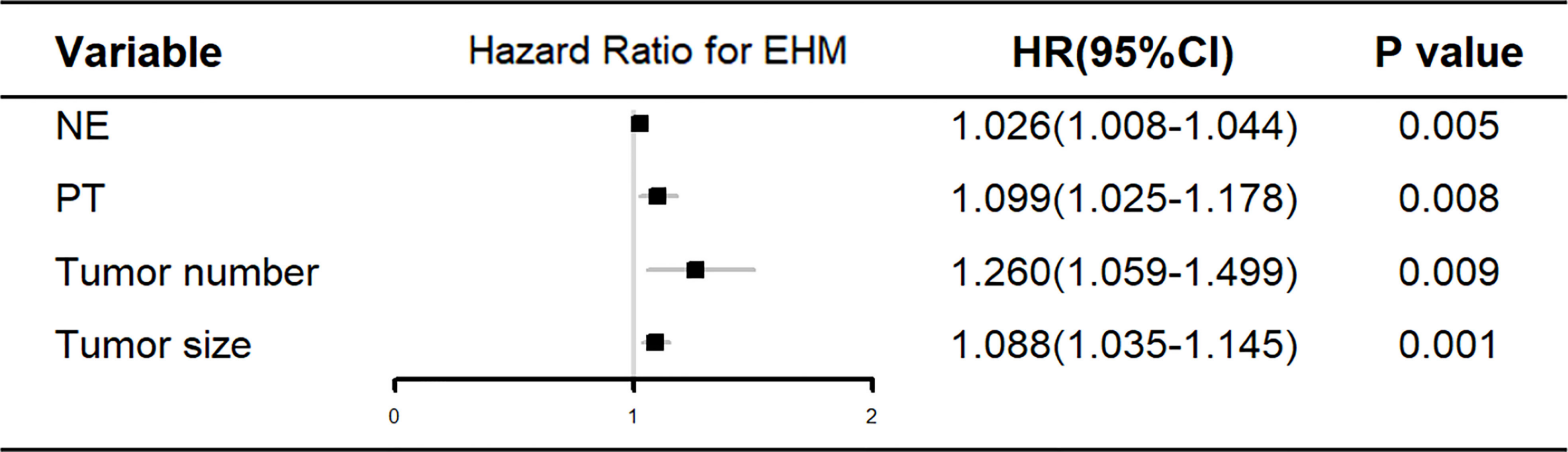
Multivariate analysis of the clinical characteristics for predicting EHM in the training cohort. NE, neutrophil; PT, prothrombin time.

The training cohort was concentrated on 136 cases in the EHM group, of which 112 (81.4%) were male and 24 (17.6%) were female, aged 53.42 (48.15, 62.69) years; 320 cases in the control group, of which 255 (79.7%) were male and 65 (20.3%) were female, aged 53.83 (46.82, 63.32) years. **Table 1** shows further characteristics of training and validation patients. According to univariate analysis and multivariate analyses, neutrophils, prothrombin time, tumor count, and size have been shown to be independent risk factors for EHM in initial patients (**Table 2** and **Figure 2**).

**Table 1:** Baseline clinical characteristics of inital patients.

Variable	Training cohort (n = 456)	Validation cohort (n = 151)
Age, years	53.69 (47.03, 63.14)	54.21 (48.04, 62.62)
Sex		
Male	367 (80.5%)	126 (83.4%)
Female	89 (19.5%)	25 (16.6%)
History of diabetes	66 (14.5%)	39 (25.8%)
History of Cirrhosis	313 (68.6%)	118 (78.1%)
History of Smoke	135 (29.6%)	57 (37.7%)
History of Drink	100 (21.9%)	44 (29.1%)
BMI	22.66 (20.57, 24.91)	23.51 (21.24, 25.80)
Child-Pugh		
A	245 (53.7%)	91 (60.2%)
B	186 (40.8%)	54 (35.8%)
C	25 (5.5%)	6 (4.0%)
WBC, ×10^9^/L	5.10 (3.80, 6.91)	4.89 (3.82, 6.42)
NE, %	65.04 (57.20, 74.50)	67.25 (59.63, 73.25)
HB, g/L	143.00 (124.00, 159.00)	146.00 (128.00, 165.25)
PLT, ×10^9^	125.00 (79.00, 182.00)	133.00 (78.50, 183.00)
ALT, U/L	67.50 (38.00, 153.50)	64.50 (44.00, 175.00)
AST, U/L	81.00 (48.00, 185.00)	102.50 (50.75, 204.50)
TP, g/L	63.40 (58.00, 68.00)	65.95 (60.58, 73.00)
ALB, g/L	33.25 (29.63, 37.00)	35.20 (31.48, 39.75)
GLO, g/L	29.50 (25.90, 33.50)	29.80 (26.88, 34.45)
TBIL, μmol/L	27.75 (17.10, 46.15)	25.10 (16.00, 43.35)
DBIL, μmol/L	11.87 (7, 20.48)	11.45 (7.28, 19.88)
ALP, U/L	144.00 (98.00, 246.85)	145.00 (96.75, 265.50)
Cr, μmol/L	58.00 (50.00, 66.13)	59.00 (49.75, 67.25)
CHE, U/L	3382.50 (2323.25, 4970.50)	4308.00 (3023.50, 5981.75)
INR	1.14 (1.03, 1.29)	1.10 (0.99, 1.21)
TT, s	19.50 (17.80, 21.00)	19.20 (17.88, 20.80)
DD, mg/L	3.60 (1.70, 7.00)	3.60 (1.50, 8.55)
APTT, s	36.10 (32.10, 44.10)	35.00 (30.05, 39.63)
FIB, g/L	3.26 (2.33, 4.26)	2.67 (2.05, 4.16)
PT, s	13.70 (12.30, 15.48)	13.15 (11.90, 14.53)
AFP, ng/ml	337.46 (13.24, 2000.00)	200.09 (20.68, 2000.00)
CEA ≥5 ng/ml	47 (10.30%)	23 (15.2%)
CA19-9 ≥35 U/ml	55 (12.10%)	12 (7.9%)
HBsAg, positive	326 (71.5%)	116 (76.8%)
Anti-HCV, positive	15 (3.3%)	1 (0.7%)
Tumor number	4 (4, >4)	4 (4, 4)
Tumor sizes, cm	7.60 (4.46, 11.14)	6.58 (4.30, 11.78)
Vascular invasion	185 (40.6%)	45 (29.8%)
Tumor Location		
Left lobe	42 (9.2%)	12 (8.0%)
Right lobe	202 (44.3%)	69 (45.7%)
Bilateral lobe	212 (46.5%)	70 (46.3%)

BMI, Body Mass Index; WBC, White Blood Cell Count; NE, Neutrophil; HB, Hemoglobin; PLT, Platelet Count; ALT, Alanine Amiotransferase; AST, Aspartate Aminotransferase; TP, Total Protein; ALB, Albumin; GLO, Globulin; TBIL, Total Bilirubin; DBIL, Direct Bilirubin; ALP, Alkaline Phosphatase; Cr, Creatinine; CHE, Cholinesterase; INR, International Normalized Ratio; TT, Thrombin Time; DD, D-Dimer; APTT, Activated Partial Thromboplastin Time; FIB, Fibrinogen; PT, Prothrombin Time; AFP, α-Fetoprotein; CEA, Carcinoembryonic Antigen; CA19-9, Carbohydrate Antigen 19-9; HBsAg, Hepatitis B Surface Antigen; Anti-HCV, Anti-Hepatitis C Virus Antibody.

**Table 2:** Univariate analysis for predicting EHM in the training cohort.

Variable	EHM group (n = 320)	Non-EHM group (n = 136)	p-Value
Age, years	53.83 (46.82, 63.32)	53.42 (48.15, 62.69)	0.959
Sex			0.511
Male	255 (79.7%)	112 (82.4%)	
Female	65 (20.3%)	24 (17.6%)	
History of diabetes	51 (15.9%)	15 (11.0%)	0.173
History of cirrhosis	219 (68.4%)	94 (69.1%)	0.886
History of smoke	94 (29.4%)	41 (30.1%)	0.869
History of drink	70 (21.9%)	30 (22.1%)	0.965
BMI	22.66 (20.57, 24.91)	22.59 (20.67, 24.83)	0.742
Child-Pugh			0.158
A	179 (55.9%)	66 (48.5%)	
B	127 (39.7%)	59 (43.4%)	
C	14 (4.4%)	11 (8.1%)	
WBC, ×10^9^/L	5.03 (3.74, 6.58)	5.37 (4.12, 7.64)	0.053
NE, %	63.70 (55.96, 73.45)	69.50 (59.03, 76.50)	0.001
HB, g/L	144.50 (126.00, 160.00)	139 (120.25, 157.00)	0.044
PLT, ×10^9^	125.00 (79.00, 177.75)	124.5 (79.50, 188.50)	0.711
ALT, U/L	63.00 (35.00, 144.50)	77.00 (43.00, 163.00)	0.031
AST, U/L	76.50 (45.00, 170.00)	104.50 (64.00, 242.00)	0.002
TP, g/L	63.30 (58.00, 68.00)	64.00 (57.70, 70.00)	0.427
ALB, g/L	33.45 (30.43, 37.00)	32.00 (28.10, 36.78)	0.039
GLO, g/L	29.00 (25.60, 32.50)	31.20 (26.33, 34.88)	0.005
TBIL, μmol/L	25.35 (16.05, 44.00)	31.60 (20.05, 52.70)	0.007
DBIL, μmol/L	11.38 (6.60, 19.08)	13.20 (8.33, 23.17)	0.038
ALP, U/L	133.00 (89.00, 217.75)	170.00 (110.25, 297.00)	0.000
Cr, μmol/L	58.00 (51.00, 66.13)	57.00 (50.00, 66.75)	0.603
CHE, U/L	3563.00 (2571.50, 5067.00)	2966.00 (2001.00, 4229.00)	0.001
INR	1.13 (1.02, 1.26)	1.18 (1.06, 1.34)	0.002
TT, s	19.6 (17.70, 21.00)	19.40 (18.00, 21.00)	0.658
DD, mg/L	3.30 (1.53, 6.78)	3.80 (2.63, 7.70)	0.019
APTT, s	35.95 (31.60, 43.20)	37.45 (32.65, 48.38)	0.066
FIB, g/L	3.21 (2.30, 4.21)	3.33 (2.41, 4.36)	0.351
PT, s	13.60 (12.20, 15.30)	14.1 (12.72, 15.98)	0.004
AFP, ng/ml	246.34 (12.16, 2000.00)	370.29 (17.92, 2000.00)	0.507
CEA ≥5 ng/ml	30 (9.38%)	17 (12.5%)	0.637
CA19-9 ≥35 U/ml	38 (11.88%)	17 (12.5%)	0.770
HBsAg, positive	231 (76.7%)	95 (72.5%)	0.348
Anti-HCV, positive	9 (3.5%)	6 (5.3%)	0.417
Tumor number	4 (2, >4)	4 (4, >4)	0.001
Tumor sizes, cm	7.00 (4.00, 10.60)	9.2 (5.91, 11.82)	0.000
Vascular invasion	115 (35.9%)	70 (51.5%)	0.002
Tumor location			0.027
Left lobe	36 (11.3%)	6 (4.4%)	
Right lobe	145 (45.3%)	57 (41.9%)	
Bilateral lobe	139 (43.4%)	73 (53.7%)	

BMI, Body Mass Index; WBC, White Blood Cell Count; NE, Neutrophil; HB, Hemoglobin; PLT, Platelet Count; ALT, Alanine Amiotransferase; AST, Aspartate Aminotransferase; TP, Total Protein; ALB, Albumin; GLO, Globulin; TBIL, Total Bilirubin; DBIL, Direct Bilirubin; ALP, Alkaline Phosphatase; Cr, Creatinine; CHE, Cholinesterase; INR, International Normalized Ratio; TT, Thrombin Time; DD, D-Dimer; APTT, Activated Partial Thromboplastin Time; FIB, Fibrinogen; PT, Prothrombin Time; AFP, α-Fetoprotein; CEA, Carcinoembryonic Antigen; CA19-9, Carbohydrate Antigen 19-9; HBsAg, Hepatitis B Surface Antigen; Anti-HCV, Anti-Hepatitis C Virus Antibody.

### Construction and validation of the initial patients EHM nomogram

**Figure 3:**
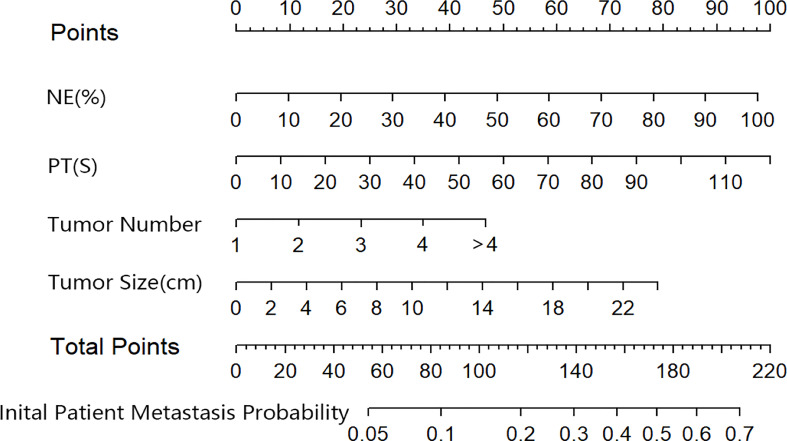
The nomogram predicting initial patient EHM probability. NE, neutrophil; PT, prothrombin time.

**Figure 4:**
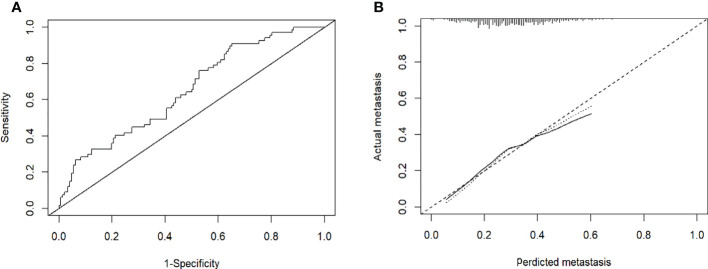
**(A)**: Receiver operating characteristic curves for EHM of the patients in the training cohort. The receiver operating characteristic curves at the initial diagnosis are shown, and its area ROC curves are provided. **(B)**: Calibration plots of EHM in the training cohorts. The calibration curves derived from the training cohorts are almost a diagonal line that would represent perfectly reliable prediction.

**Figure 5:**
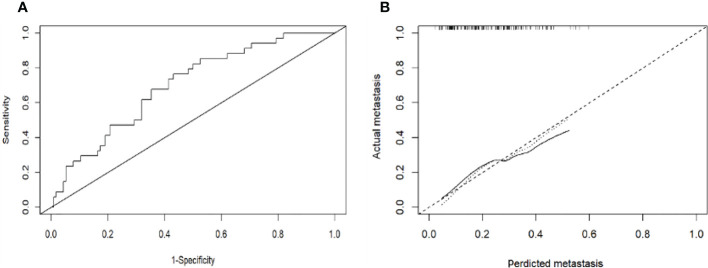
**(A)**: Receiver operating characteristic curves for EHM of the patients in the validation cohort. The receiver operating characteristic curves at the initial diagnosis are shown, and its area ROC curves are provided. **(B)**: Calibration plots of EHM in the validation cohort. The calibration curves derived from the validation cohort are almost a diagonal line that would represent perfectly reliable prediction.

Based on the results of the multivariate logistic regression analysis in the training cohort, neutrophil, prothrombin time, tumor number, and tumor size were used as variables to construct the nomogram (**Figure 3**). When the ROC curve was plotted using the training cohort, the area under the ROC curve was calculated as 0.672 (**Figure 4A**). In the validation cohort, the nomogram displayed a C index of 0.694 (**Figure 5A**). This result indicates that there is some discrimination in the mode and can be relied on to accurately predict the extrahepatic metastases of hepatocellular carcinoma. Model consistency was assessed by drawing calibration curves using data from the Training and Validation cohorts (**Figures 4B**, **5B**). The diagonal line represents the precise match between the expected and real circumstances, the dashed line represents the model’s theoretical forecast, and the solid line represents the actual prediction obtained by repeated sampling. The two curves are less discontinuous from the diagonal line, suggesting that the model-anticipated results are more consistent with what actually occurred and the model-predicted results are more credible.

### Follow-up

**Figure 6:**
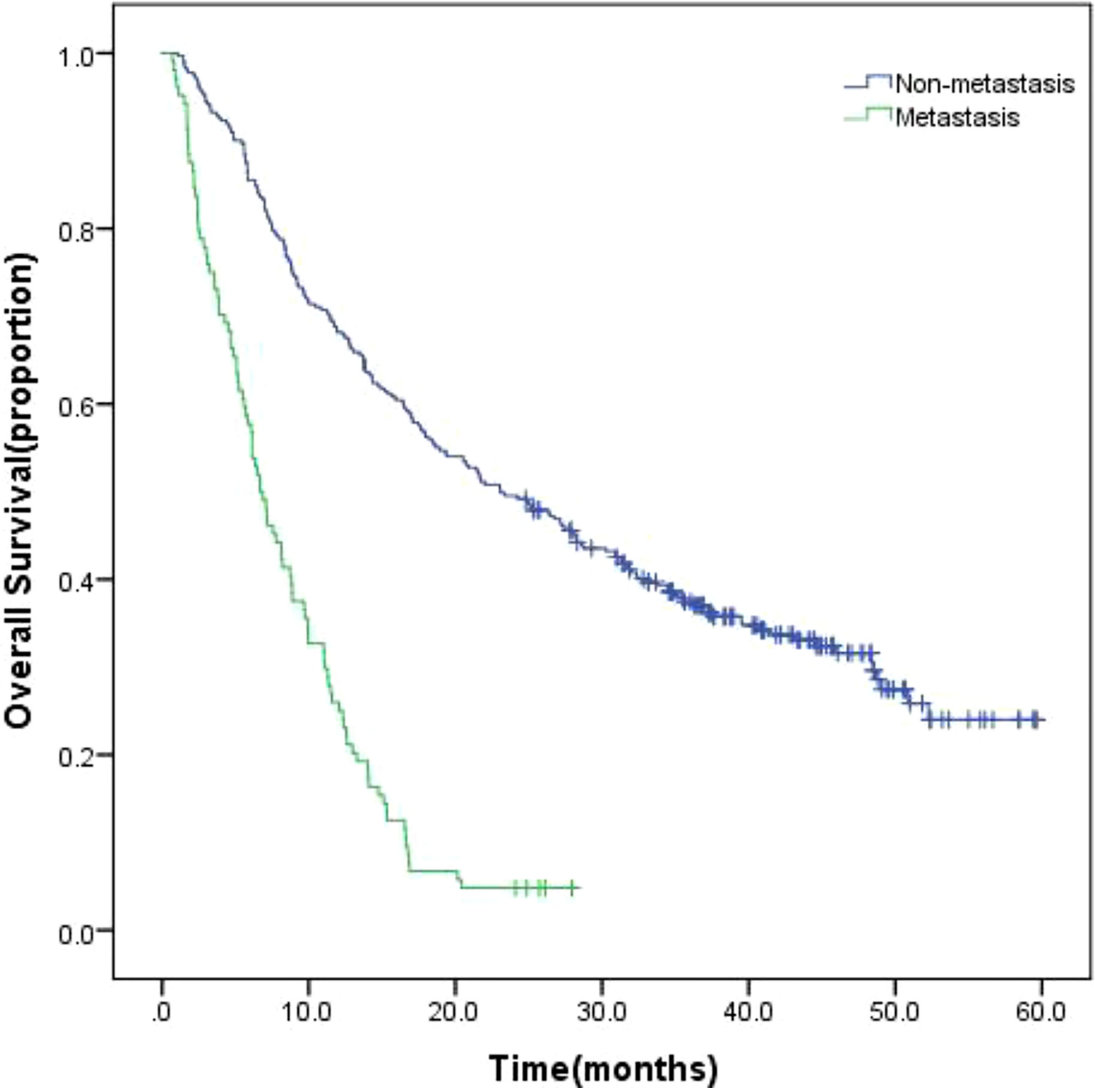
Kaplan-Meier survival curves of all enrolled patients.

The 415 patients were fully monitored for 23.694 person-months (median, 15.118 months; range, 3.7 to 62.6 months), in which 308 (74.2%) died. In all 415 patients enrolled, the overall survival (OS) of patients with EHM was significantly worse than non-EHM patients (**Figure 6**, 6.7 months *vs*. 23.1 months, p = 0.00).

A total of 372 patients were enrolled in this study without metastasis at the time of initial diagnosis and all were followed up. A total of 372 patients were enrolled in this study without metastasis at the time of initial diagnosis, all of whom underwent follow-up. Based on the EHM occurring during the follow-up period, the patients were divided into observational metastases and non-methane groups. The characteristics of the patients are listed in **Table 3**. According to univariate analysis and multivariate analyses, neutrophils, Total Protein, Carcinoembryonic Antigen and tumor sizes have been shown to be independent risk factors for EHM in followed up patients (**Table 4**).

**Table 3:** Baseline clinical characteristics of followed up patients.

Variable	EHM group (n = 282)	Non-EHM group (n = 90)
Age, years	54.62 (47.26,63.37)	53.12 (47.93,62.67)
Sex		
Male	228 (80.85%)	66 (73.33%)
Female	54 (19.15%)	24 (26.67%)
History of diabetes	46 (16.31%)	23 (25.56%)
History of cirrhosis	202 (71.63%)	62 (68.89%)
History of smoke	87 (30.85%)	32 (35.56%)
History of drink	65 (23.05%)	24 (26.67%)
BMI	23.04 (20.83, 25.34)	22.49 (20.24, 25.44)
Child-Pugh		
A	158 (56.03%)	65 (72.22%)
B	113 (40.07%)	24 (26.67%)
C	11 (3.90%)	1 (1.11%)
WBC, ×10^9^/L	4.80 (3.62, 6.37)	5.14 (3.84, 6.86)
NE, %	62.87 (55.73, 72.10)	63.55 (56.235, 72.55)
HB, g/L	144.00 (125.75, 161.00)	144.50 (126.25, 164.00)
PLT, ×10^9^	124.50 (71.00, 178.75)	132.00 (83.75, 193.50)
ALT, U/L	66.00 (37.00, 143.00)	60.00 (37.75, 174.00)
AST, U/L	78.50 (45.00, 180.25)	63.50 (44.75, 151.75)
TP, g/L	64.80 (59.48, 68.73)	63.45 (58.68, 68.55)
ALB, g/L	33.75 (30.30, 38.20)	34.40 (31.28, 38.53)
GLO, g/L	29.25 (26.18, 33.30)	28.70 (24.98, 32.68)
TBIL, μmol/L	26.50 (16.55, 45.33)	19.50 (13.43, 31.10)
DBIL, μmol/L	11.50 (7.13, 20.18)	9.25 (5.48, 14.68)
ALP, U/L	128.85 (88.85, 224.13)	135.45 (90.50, 229.40)
Cr, μmol/L	59.00 (51.00, 67.50)	54.00 (48.00, 64.00)
CHE, U/L	3816.00 (2625.00, 5451.50)	3946.00 (3172.00, 5356.00)
INR	1.11 (1.01, 1.25)	1.07 (0.98, 1.21)
TT, s	19.50 (17.90, 21.00)	19.00 (17.40, 20.80)
DD, mg/L	3.10 (1.50, 6.80)	3.60 (1.50, 6.05)
APTT, s	35.65 (30.90, 43.40)	34.80 (30.90, 40.50)
FIB, g/L	2.94 (2.21, 4.02)	3.62 (2.36, 4.80)
PT, s	13.40 (12.10, 15.03)	12.80 (11.80, 14.55)
AFP, ng/ml	112.21 (11.59, 2000.00)	440.33 (25.91, 2000.00)
CA19-9 ≥35 U/ml	29 (10.28%)	13 (14.44%)
CEA ≥5 ng/ml	33 (11.70%)	9 (10.00%)
HBsAg, positive	211 (74.82%)	62 (68.89%)
Anti-HCV, positive	8 (2.84%)	1 (1.11%)
Tumor number	4 (2, 4)	4 (4, >4)
Tumor sizes, cm	5.96 (3.68, 9.91)	7.59 (4.55, 11.18)
Vascular invasion	100 (35.46%)	24 (26.67%)
Tumor location		
Left lobe	35 (12.41%)	6 (6.67%)
Right lobe	133 (47.16%)	36 (40.00%)
Bilateral lobe	114 (40.43%)	48 (53.33%)

BMI, Body Mass Index; WBC, White Blood Cell Count; NE, Neutrophil; HB, Hemoglobin; PLT, Platelet Count; ALT, Alanine Amiotransferase; AST, Aspartate Aminotransferase; TP, Total Protein; ALB, Albumin; GLO, Globulin; TBIL, Total Bilirubin; DBIL, Direct Bilirubin; ALP, Alkaline Phosphatase; Cr, Creatinine; CHE, Cholinesterase; INR, International Normalized Ratio; TT, Thrombin Time; DD, D-Dimer; APTT, Activated Partial Thromboplastin Time; FIB, Fibrinogen; PT, Prothrombin Time; AFP, α-Fetoprotein; CEA, Carcinoembryonic Antigen; CA19-9, Carbohydrate Antigen 19-9;HBsAg, Hepatitis B Surface Antigen; Anti-HCV, Anti-Hepatitis C Virus Antibody.

**Table 4:** Univariate and multivariate analysis for predicting EHM in followed up patients.

Variable	Univariate analysis			Multivariate analysis		
	HR	95%CI	p-Value	HR	95%CI	p-Value
Age, years	0.999	0.978-1.021	0.951			
Sex						
Male						
Female	1.452	0.901–2.341	0.126			
History of diabetes	0.893	0.549–1.452	0.647			
History of cirrhosis	0.676	0.428–1.066	0.092			
History of smoke	0.831	0.537–1.285	0.405			
History of drink	0.756	0.473–1.211	0.245			
BMI	0.978	0.915–1.046	0.517			
Child–Pugh						
A	0.256	0.034–1.913	0.184			
B	0.300	0.039–2.289	0.245			
C						
WBC, ×10^9^/L	1.063	0.970–1.164	0.192			
NE, %	1.028	1.009–1.048	0.004	1.021	1.000–1.044	0.044
HB, g/L	1.000	0.994–1.006	0.930			
PLT, ×10^9^	1.002	0.999–1.005	0.187			
ALT, U/L	1.000	1.000–1.001	0.245			
AST, U/L	1.001	1.000–1.001	0.093			
TP, g/L	0.975	0.950–1.001	0.057	0.970	0.942–0.998	0.039
ALB, g/L	0.975	0.932–1.020	0.267			
GLO, g/L	0.975	0.944–1.007	0.123			
TBIL, μmol/L	1.002	0.997–1.007	0.376			
DBIL, μmol/L	1.003	0.997–1.009	0.331			
ALP, U/L	1.001	1.000–1.003	0.117			
CHE, U/L	1.000	1.000–1.000	0.765			
INR	1.009	0.970–1.049	0.663			
TT, s	0.997	0.936–1.063	0.932			
DD, mg/L	1.012	0.993–1.031	0.224			
APTT, s	1.014	0.984–1.044	0.368			
FIB, g/L	1.102	0.952–1.277	0.194			
PT, s	1.015	0.922–1.117	0.765			
AFP, ng/ml	1.000	1.000–1.000	0.309			
CA19-9 ≥35 U/ml	1.000	0.999–1.001	0.917			
CEA ≥5 ng/ml	6.240	2.832–13.747	0.000	1.281	1.100–1.491	0.001
HBsAg, positive	1.290	0.796–2.091	0.302			
Anti-HCV, positive	0.273	0.037–2.016	0.203			
Tumor number	1.049	0.892–1.235	0.562			
Tumor sizes, cm	1.083	1.032–1.136	0.001	1.072	1.014–1.134	0.014
Vascular invasion	1.705	1.051–2.764	0.031			
Tumor location						
Left lobe	1.294	0.547–3.059	0.558			
Right lobe	0.939	0.606–1.454	0.778			
Bilateral lobe						

BMI, Body Mass Index; WBC, White Blood Cell Count; NE, Neutrophil; HB, Hemoglobin; PLT, Platelet Count; ALT, Alanine Amiotransferase; AST, Aspartate Aminotransferase; TP, Total Protein; ALB, Albumin; GLO, Globulin; TBIL, Total Bilirubin; DBIL, Direct Bilirubin; ALP, Alkaline Phosphatase; Cr, Creatinine; CHE, Cholinesterase; INR, International Normalized Ratio; TT, Thrombin Time; DD, D-Dimer; APTT, Activated Partial Thromboplastin Time; FIB, Fibrinogen; PT, Prothrombin Time; AFP, α-Fetoprotein; CEA, Carcinoembryonic Antigen; CA19-9, Carbohydrate Antigen 19-9;HBsAg, Hepatitis B Surface Antigen; Anti-HCV, Anti-Hepatitis C Virus Antibody.

## Discussion

The incidence of HCC has increased in many countries in recent years. The primary risk factors for HCC worldwide include chronic hepatitis B virus (HBV) or hepatitis C virus (HCV) and the consumption of aflatoxin-contaminated food. The prevalence of HCC caused by metabolic syndrome, obesity, diabetes, excessive alcohol consumption, and non-alcoholic fatty liver disease (NAFLD) is gradually increasing (8). Thus, as the etiology of the disease has changed, the risk factors for EHM of HCC may also be changed, and further research is required.

For patients with HCC with EHM, most previous studies examined only the relationship between clinicopathological characteristics and the prognosis of patients with HCC (5, 9–11). However, the common shortcoming was that the patients enrolled previously received anti-tumor treatment, and the lab results were incidentally altered (to exhibit low white blood cell counts, low platelet counts, poor liver or kidney function, and so on) (9), which may bring inevitable interference to the analysis of metastases. Therefore, this study investigated the clinical characteristics and risk factors of patients with HCC with EHM who did not receive anti-tumor treatment, and established an effective diagnostic nomogram for EHM. In our study, 28% (170) of all 607 HCC patients included had extrahepatic metastases, consistent with previous study results (9, 12). In the initially diagnosed patients, the metastatic sites included the lymph node (122, 71.8%), lung (104, 61.2%), bone (12, 7.1%), diaphragm (7, 4.1%), peritoneum (4, 2.3%), spleen (3, 1.8%), pancreas (2, 1.2%), adrenal gland (2, 1.2%), and stomach (1, 0.6%). The lung may be the most common site of EHM from HCC speciously, but in our study, the proportion of lymph node metastases was the highest (122, 71.8%). Additionally, a similar finding was obtained by another Chinese study (13). In that research study, among the 132 patients with extrahepatic metastases from hepatocellular carcinoma diagnosed by whole-body PET/CT, 72 (54.5%) had metastases in the lymph node, 32 (24.2%) had metastases in the bones, and 28 (21.2%) had metastases in the lungs. This may due to the main symptom of patients with simple lymph node metastases rarely present with clinical symptoms, and only a small proportion of patients are noted when the enlarged lymph nodes caused a compression effect, for example, the jaundice caused by bile duct compression. As a result, the rate of missed lymph node metastases was high and easily ignored in a relatively earlier stage of metastasis.

According to the univariate and multivariate analysis of EHM in our research (**Table 2** and **Figure 2**), there may be a potential relation between HCC patients with EHM and the tumor number. This relationship was also mentioned in another report which showed that the number of tumors >2 can be easier found in patients with EHM (14). They therefore concluded that the number of tumors could be associated with aggressive biological features. Coincidentally, in our research, the tumor counts are independent predictors for EHM (**Figure 2**). Similar findings for HCC metastasis were noted previously, which revealed that a multiple tumor number was a risk factor for EHM in HCC (15, 16).

A close relationship between HCC size and EHM seems to exist according to our results (**Figure 2**). One possible explanation is that the lager lesion contains more tumor stem cells, which are frequently identified as the source of malignant phenotypes such as aggressive growth, portal vein thrombosis, or metastasis. Another explanation is that the tumor biology changes beyond a certain mass, just as tumors in general cannot grow beyond a critical small size (9, 17, 18).

In this study, we also demonstrated that an elevated neutrophil was a significant independent predictor for EHM of HCC. Neutrophil is one of the most simple and effective markers of inflammation and is associated with poor prognosis in various cancers (11, 19, 20). Therefore, the immanent reason may be that the high neutrophil count is a symbol of an adequate environment for tumor progression, which has been shown to promote tumor growth and metastasis by secreting chemokines, vascular endothelial growth factor, and matrix metalloproteinase-9, which are involved in the development of local inflammation and angiogenesis (21–23).

Currently, angiopoietin-2 (Ang-2), microRNAs (miRNAs), and lncRNA BACE1-AS are available as a test for the evaluation of EHM in HCC (24–27). However, the above parameters are mainly laboratory-based and are not available in most hospitals. Therefore, our results identified the risk factors for EHM of HCC which are based on noninvasively clinically readily available data and developed a nomogram with some predictive ability. We constructed a predictive model of EHM of HCC by taking the above independent risk factors as variables. It is used to predict the initial probability of patient EHM, and validated internally and externally, confirming its certain predictive capacity for EHM. The calibration curves were drawn and showed that the nomogram predictions overlapped well with the actual clinical situation, with good agreement and credible prediction results.

In previous studies (9, 12, 14, 28), most researchers focused on the observation of the patient’s prognosis. Therefore, only a preliminary analysis of the reference indicators associated with EHM was performed. This study further explored the independent risk factors for EHM and developed a nomogram with some predictive ability, building on the previous work. It has a role to play in reducing missed EHM and designing optimal therapies for those patients. This study is a single-center study only and the model could be further improved with a larger sample size and multi-center data.

## Data availability statement

The original contributions presented in the study are included in the article/supplementary material. Further inquiries can be directed to the corresponding authors.

## Ethics statement

The study was censored on October 19,2020 and was approved by the Ethics Committee of The Affiliated Hospital of Qinghai University. All subjects signed an informed consent form. The patients/participants provided their written informed consent to participate in this study.

## Author contributions

LZ is responsible for writing, LR, MQ, WW and XS are responsible for document of patients data. FY, MD and HW are responsible for document. following up, ZW, HF are responsible for guiding research and revising papers. All authors contributed to the article and approved the submitted version.

## Funding

This study was supported by the National Natural Science Foundation of China (No. 82160466) and Research team for minimally invasive diagnosis and treatment of biliary and pancreatic diseases (The Affiliated Hospital of Qinghai University).

## Conflict of interest

The authors declare that the research was conducted in the absence of any commercial or financial relationships that could be construed as a potential conflict of interest.

## Publisher’s note

All claims expressed in this article are solely those of the authors and do not necessarily represent those of their affiliated organizations, or those of the publisher, the editors and the reviewers. Any product that may be evaluated in this article, or claim that may be made by its manufacturer, is not guaranteed or endorsed by the publisher.
